# Molecular Features of HHV8 Monoclonal Microlymphoma Associated with Kaposi Sarcoma and Multicentric Castleman Disease in an HIV-Negative Patient

**DOI:** 10.3390/ijms25073775

**Published:** 2024-03-28

**Authors:** Evelina Rogges, Sabrina Pelliccia, Camilla Savio, Gianluca Lopez, Irene Della Starza, Giacinto La Verde, Arianna Di Napoli

**Affiliations:** 1 Department of Medical and Surgical Sciences and Translational Medicine, Faculty of Medicine and Psychology, PhD School in Translational Medicine and Oncology, Sapienza University of Rome, 00189 Rome, Italy; evelina.rogges@uniroma1.it; 2 Hematology Unit, Department of Clinical and Molecular Medicine, Sant’Andrea University Hospital, Sapienza University of Rome, 00189 Rome, Italy; sabrina.pelliccia@uniroma1.it (S.P.); giacinto.laverde@ospedalesantandrea.it (G.L.V.); 3 Medical Genetics Unit, Department of Diagnostic Sciences, Sant’Andrea University Hospital, 00189 Rome, Italy; camilla.savio@ospedalesantandrea.it; 4 Pathology Unit, Department of Clinical and Molecular Medicine, Sant’Andrea University Hospital, Sapienza University of Rome, 00189 Rome, Italy; gianluca.lopez@ospedalesantandrea.it; 5 Hematology, Department of Translational and Precision Medicine, Sapienza University of Rome, 00161 Rome, Italy; dellastarza@bce.uniroma1.it

**Keywords:** HHV8, MCD, microlymphoma, HHV8+ DLBCL, extra-cavitary PEL, mutations, somatic hypermutation

## Abstract

Human herpesvirus 8 (HHV8)-associated diseases include Kaposi sarcoma (KS), multicentric Castleman disease (MCD), germinotropic lymphoproliferative disorder (GLPD), Kaposi sarcoma inflammatory cytokine syndrome (KICS), HHV8-positive diffuse large B-cell lymphoma (HHV8+ DLBCL), primary effusion lymphoma (PEL), and extra-cavitary PEL (ECPEL). We report the case of a human immunodeficiency virus (HIV)-negative male treated for cutaneous KS, who developed generalized lymphadenopathy, hepatosplenomegaly, pleural and abdominal effusions, renal insufficiency, and pancytopenia. The excised lymph node showed features of concomitant involvement by micro-KS and MCD, with aggregates of HHV8+, Epstein Barr virus (EBV)-negative, IgM+, and lambda+ plasmablasts reminiscent of microlymphoma. Molecular investigations revealed a somatically hypermutated (SHM) monoclonal rearrangement of the immunoglobulin heavy chain (IGH), accounting for 4% of the B-cell population of the lymph node. Mutational analyses identified a pathogenic variant of *KMT2D* and variants of unknown significance in *KMT2D*, *FOXO1*, *ARID1A*, and *KMT2A*. The patient died shortly after surgery. The histological features (HHV8+, EBV−, IgM+, Lambda+, MCD+), integrated with the molecular findings (monoclonal IGH, SHM+, *KMT2D* mutated), supported the diagnosis of a monoclonal HHV8+ microlymphoma, with features intermediate between an incipient HHV8+ DLBCL and an EBV-negative ECPEL highlighting the challenges in the accurate classification of HHV8-driven lymphoid proliferations.

## 1. Introduction

Kaposi sarcoma herpesvirus/human herpesvirus 8 (KSHV/HHV8) is etiologically related to a spectrum of unique clinicopathologic entities mostly occurring in patients with an immunodeficiency [1].

HHV8-associated multicentric Castleman’s disease (HHV8-MCD) is a subtype of MCD, which comprises a heterogeneous group of lymphoproliferative disorders characterized by lymphadenopathies and systemic inflammatory symptoms driven by the excessive production of interleukins (IL), especially IL-6 [2]. HHV8-MCD mostly affects immunocompromised patients, particularly in association with HIV infection, but it can also affect immunocompetent individuals, mainly in HHV8-endemic areas. Histologically, HHV8-infected large cells resembling immunoblasts or plasmablasts are scattered in the expanded mantle zones of atrophic or hyperplastic follicles in a lymph node, with interfollicular plasmacytosis and increased vascularization [1,2,3]. HHV8-infected cells express MUM1, IgM, and lambda light chain, variably CD20 and CD79a, and show polyclonal unmutated immunoglobulin genes (IG), suggesting their origin from naïve B-cells. They may expand and aggregate into small sheets, previously termed “microlymphoma” [2,3,4,5,6], with the potential to progress to a monoclonal proliferation and then to an overt HHV8-positive diffuse large cell B-cell lymphoma (HHV8+ DLBCL) [7].

In HHV8+ DLBCL, monoclonal HHV8/IgM/Lambda plasmablasts efface the lymph node architecture and should be differentiated from an extra cavitary-primary effusion lymphoma (EC-PEL), which may occur as well in patients with MCD. In EC-PEL the neoplastic effusion in body cavities characteristic of PEL is lacking. Unlike HHV8+ DLBCL, the malignant cells in PEL/ECPEL are usually negative for pan–B-cell antigens, express MUM1, CD138, frequently CD30, and monotypic kappa or lambda light chains in about 40% of the cases [8]. They also usually show co-infection by Epstein Barr virus (EBV) and have hypermutated IG, indicating they derive from antigen-experienced B-cells [9,10].

Germinotropic lymphoproliferative disorder (GLPD) is a polyclonal proliferation of MUM1+ plasmablasts coinfected by Epstein Barr virus (EBV) and HHV8, which expand within the germinal centers of lymph nodes with no pathological features of MCD. It usually occurs in elderly, immunocompetent patients [1,3].

Kaposi sarcoma inflammatory cytokine syndrome (KICS) is an aggressive disorder occurring in HIV+ or transplanted patients with concurrent Kaposi sarcoma (KS) (90% of the cases) and/or PEL (20% of the cases). Similarly to HHV8-MCD, KICS is associated with an overproduction of human IL-6 and IL-10 and viral IL-6, and clinically manifests with fever, dyspnea, weight loss, fluid retention, lymphadenopathies, splenomegaly, hepatomegaly, and with an elevated serum HHV8 viral load but without histological evidence of MCD [11,12].

Kaposi sarcoma (KS) is a low-grade vascular tumor and an AIDS-defining illness caused by the infection and proliferation of endothelial cells of blood and lymphatic vessels [13]. The co-existence of KS and KICS or MCD in the same lymph node is not uncommon [11,14,15], but only a few cases have been reported in HIV-negative patients [16,17,18].

Herein, we describe the case of an HIV-negative man affected by cutaneous KS with simultaneous lymph node involvement by KS, HHV8-MCD, and a monoclonal HHV8+ microlymphoma. In this setting, we provide the first comprehensive molecular characterization, through next-generation sequencing (NGS), of immunoglobulin heavy chain (IGH) and 54 lymphoma-related genes. The relevant literature on HHV8 microlymphoma is also herein reviewed.

## 2. Detailed Case Description 

### 2.1. Clinical Data

A 66-year-old Caucasian male with a diagnosis of cutaneous Kaposi sarcoma (KS), previously treated with gemcitabine chemotherapy for two months, was referred to the Intensive Care Unit (ICU) of our hospital for severely worsening fatigue, dyspnea, and pancytopenia. The patient was HIV-negative, had no history of systemic diseases, drug abuse, or long-term use of steroids and did not show signs or symptoms compatible with an autoimmune pathology. There were no recurring infections noted in the medical history, nor were there alterations in previous hematological and biochemical tests. The patient had well-controlled type II diabetes mellitus with oral hypoglycemic agents. The serum protein electrophoresis did not show any deficiency in gamma globulins, and lymphocyte typing did not reveal any notable alterations. Blood tests showed leukocytosis (15.3 × 10^3^/μL), anemia (hemoglobin 7.5 g/dL), thrombocytopenia (83 × 10^3^/μL), and elevated levels of the C-reactive protein (12.40 mg/dL) and of the erythrocyte sedimentation rate (59 mm/h), whereas procalcitonin was within the normal range. A polymerase chain reaction showed high titers (>50,000 copies/mL) of HHV8 DNA in peripheral blood mononuclear cells. A physical examination showed bilateral axillary and inguinal lymphadenopathy without skin lesions. A computed tomography (CT) scan showed diffuse bilateral interstitial peribronchovascular thickening, atelectasis of the lower lungs, bilateral pleural effusion, hepatosplenomegaly, massive ascites, and axillary, mediastinal, intraabdominal, pelvic, and bilateral inguinal lymphadenopathy (maximum diameter 3 cm). Oxygen saturation was 85%, with no benefit from oxygen integration with a high-flow nasal cannula (HFNC), and a worsening renal insufficiency (blood urea nitrogen 74 mg/dL and creatinine 3.43 mg/dL) implied the need for dialysis support. Bone marrow aspiration revealed a marked hypocellular bone marrow with trilinear hypoplasia. Intravenous treatment with ganciclovir was started (5 mg/kg two times a day). To evaluate the hypothesis of an HHV8-positive lymphoproliferative disorder, an axillary lymph node excisional biopsy was performed. Unfortunately, the patient’s clinical condition deteriorated rapidly within a few weeks, leading to multiorgan failure and death.

### 2.2. Histological Findings

**Figure 1:**
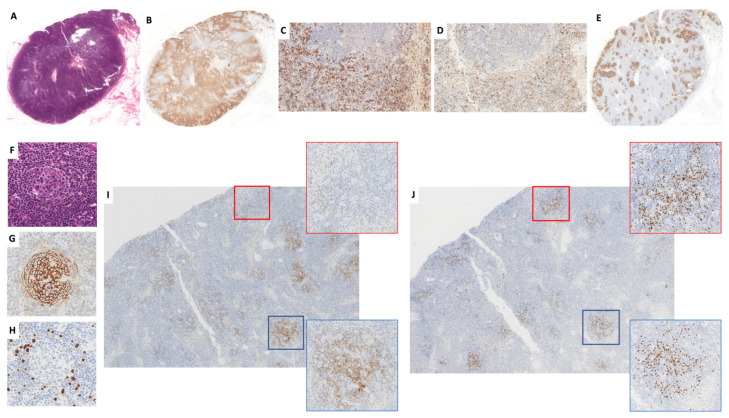
Lymph node ((**A**): H&E, o.m. ×7) with marked interfollicular polytypic plasmacytosis ((**B**): CD138, o.m. ×7; (**C**): Lambda o.m. ×260; (**D**): kappa, o.m. ×260) and B-cell nodules (**E**): CD20, o.m ×7), with rare recognizable atrophic follicles ((**F**): H&E, o.m. ×400), dense FDC meshworks ((**G**): CD23, o.m. ×400), and scattered HHV8+ plasmablasts in the mantle zones ((**H**): LANA1, o.m. ×400). The majority of the B-cell nodules showed remnants of (blue square) or absent (red square) FDC meshworks ((**I**): CD21, o.m. ×25, inserts ×160) and contained aggregates of HHV8-infected cells ((**J**): LANA1, o.m 25, inserts ×160).

A lymph node biopsy showed increased vascularity and prominent polytypic plasmacytosis encircling the CD20-positive B-cell nodules, some of which were characterized by a disrupted CD23+ and CD21+ follicular dendritic cell (FDC) meshwork and scattered IgD+ cells, reminiscent of lymphoid follicles. Rare atrophic follicles made by compact dense networks of FDCs and rare BCL6+, CD10−/+, and BCL2− cells were also present (Figure 1A–J).

**Figure 2:**
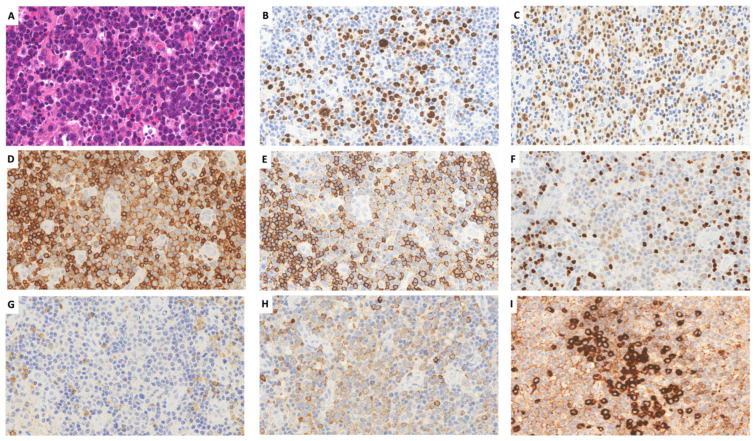
Large cells with prominent nucleoli resembling plasmablasts ((**A**): H&E) and expressing LANA1 (**B**), MUM1 (**C**), CD79a (**D**), partially CD20 (**E**), and weakly PAX5 (**F**). The majority were lambda-positive ((**G**): kappa, (**H**): lambda), whereas a proportion were IgM+ (**I**) (all images, o.m. ×400).

Large cells negative for CD138 and CD30 but expressing LANA-1, MUM1, IgM, and lambda, and variably positive for CD20 and CD79a, were sparse within the mantle zones of the atrophic follicles and formed larger aggregates within the B-cell nodules (Figure 1H,J and Figure 2A–I). In situ hybridization for EBERs showed rare positive cells in the perifollicular areas where small mature CD3+ T-cells were present. The proliferation index was high in the interfollicular regions (Ki-67 = 60%) and lower in the follicles (Ki-67 = 15%).

**Figure 3:**
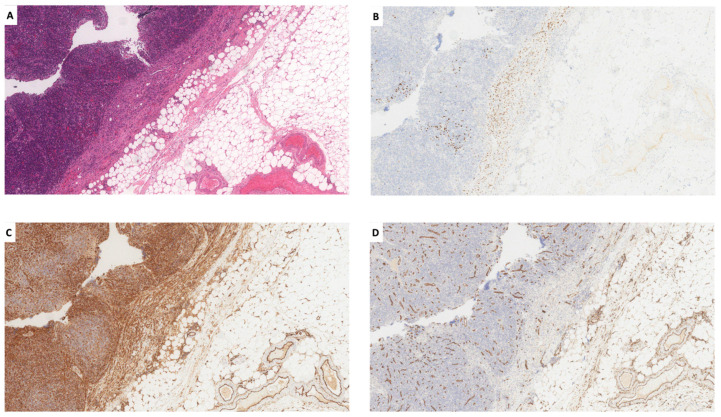
Lymph node capsule with slit-like vascular proliferation ((**A**): H&E) composed of endothelial cells positive for LANA1 (**B**), and CD31 (**C**), but negative for CD34 (**D**) (all images o.m. ×53).

These findings excluded the diagnosis of KICS, favoring HHV8-MCD with foci of microlymphoma. In addition, in a thickened area of the lymph node capsule, a LANA1+, CD34−, CD31+, slit-like vascular proliferation, suggestive of lymph node involvement by a microscopic KS, was observed (Figure 3A–D).

### 2.3. Clonality Analyses

**Figure 4:**
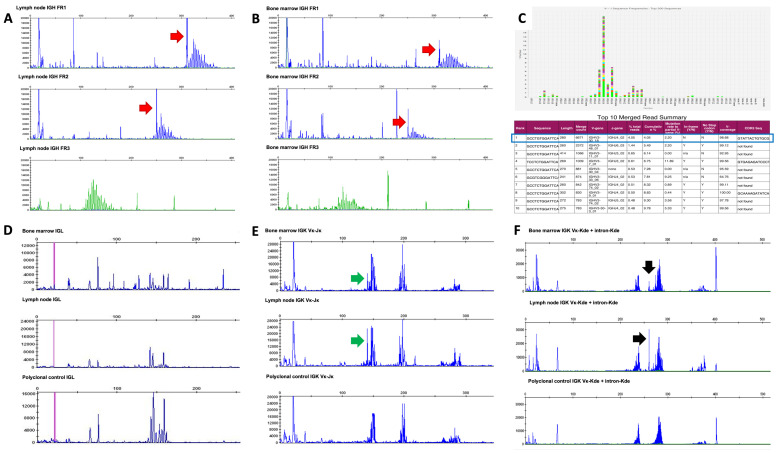
Clonality studies showed the presence of an identical monoclonal rearrangement (red arrows) on a polyclonal background of the IGH gene in the FR1 and FR2 mixes on both the lymph node (**A**) and the bone marrow blood (**B**), which accounted for 4.05% of the total reads by IGH FR1 NGS analysis with a somatic hypermutation rate of 2.2% ((**C**): green part of the column in the upper panel and blue rectangle in the lower panel). Gene scan analyses detected a polyclonal IGL gene rearrangement (**D**), whereas the IGK gene (**E**,**F**) showed a polyclonal rearrangement with a mild prevalence of a peak in the Vκ-Jκ PCR mix in both the bone marrow and the lymph node ((**E**), green arrows). Another prominent peak with a molecular weight of 260 bp that was outside the reference ranges was observed in the Vκ-Kde + intron-Kde PCR mix ((**F**), black arrows).

Clonality studies, performed using a gene scan polymerase chain reaction approach (IdentiClone IGH geneclonality, InVivoScribe Inc., San Diego, CA, USA) on the DNA extracted from the lymph node and the bone marrow (BM) blood, showed the same monoclonal IGH rearrangement on a polyclonal background in both sites (Figure 4A,B). The framework region 1 (FR1) of the IGH gene was further investigated by next-generation sequencing (NGS) analysis (LymphoTrack Dx FR1 IGH Assay, InVivoScribe Inc., on an Illumina MiSeq platform) as previously described [19]. The following guidelines for the NGS clonality assays were applied [20]: (1) a clonal rearrangement is defined by the presence of a specific clonotype in ≥2.5% of total reads of the merged rearrangement sequences; and (2) a clonal rearrangement should be ≥3 times the percentage reads of the third top-merged sequence. The analysis found a prevalent productive IGH rearrangement (VH3-30_18—JH4_02), accounting for 4.05% of the total number of reads (total read count: 164,746), showing a 2.2% somatic hypermutation (SHM) (Figure 4C).

PCR fragment and NGS clonality testing are generally highly concordant, although a linear equivalence between the height of the peak revealed by gene scan analysis and the percentage of the clonotypes detected by NGS is not always observed. This is because different PCR fragments may show the same size (base pairs), despite having different VDJ rearranged sequences [21,22]. In this light, the prominent monoclonal peak we detected in the lymph node by the PCR fragment assay likely comprised the 4% clonal rearrangement detected by NGS analysis.

PCR gene scan analyses for the light-chain genes were also performed on the lymph node and the BM blood (IdentiClone IGK and IGL geneclonality, InVivoScribe Inc.). Unexpectedly, the IGL was polyclonal (Figure 4D), whereas the IGK was polyclonal with a mild prominentf peak, with the same molecular weight in both the lymph node and the bone marrow blood (green arrow Figure 4E). A second prominent peak with a molecular weight of 260 bp was detected, although it was outside the reference ranges (210–250 bp, 270–300 bp, 350–390 bp) (Figure 4F).

All together, these findings suggested the progression of the microymphoma into a monoclonal lymphoproliferative disorder, at the same time raising the issue of a differential diagnosis between an incipient HHV8+ DLBCL and a limited lymph node involvement by an EBV-neg ECPEL.

### 2.4. Mutational Analyses

Since malignant transformation could be favored by the accumulation of molecular alterations, and molecular data regarding HHV8 microlymphomas are lacking, we performed a targeted NGS analysis on the DNA extracted from the lymph node, interrogating 54 genes frequently mutated in B-cell malignancies (SOPHIA DDM^TM^ panel for Lymphoid Malignancies on an Illumina Nextseq550 platform). The percentage of target regions with a coverage of 500× and 1000× was 100% and 99.99%, respectively, and the coverage 10% quantile was 8785×. The data analysis, conducted using Sophia DDMTM software (version 5.10.45—b296118-3685034, SOPHIA GENETICS, Rolle, Switzerland), identified a pathogenic frameshift mutation in *KMT2D* and other missense variants of uncertain significance (VUS) in *KMT2D*, *KMT2A*, *FOXO1*, and *ARID1A* (Table 1). No copy number alterations were found in the 54 investigated genes.

## 3. Discussion

HHV8 is thought to be responsible for the differentiation into plasmablasts of IgM-positive naïve B-cells in the absence of germinal center reaction [5,6] and for their restriction of the lambda light chain by inducing human immunoglobulin editing (B-cell receptor revision) in mature kappa-expressing lymphocytes [3,23,24]. Its role in lymphomagenesis has been linked to the expression of latent and lytic cycle proteins [3]. In particular, viral cyclin (vCYC) induces S-phase entry and cell proliferation; viral FLICE-inhibitory protein (vFLIP) induces the expression of antiapoptotic proteins and pro-inflammatory cytokines; latency-associated nuclear antigen (LANA) inactivates the tumor-suppressor proteins p53 and RB1 and upregulates the oncogene MYC; Kaposin B activates mitogen-activated protein kinase–associated protein kinase 2 (MAPKAPK2) and prolongs the half-lives of MYCs; viral interferon regulatory factor 3 (vIRF3) inactivates p53; and KSHV microRNAs inhibit apoptosis and promote viral latency. Despite the activity of its oncogenic proteins, HHV8 is not able to transform B-cells in culture [25], and a long latency preceded the development of B-cell lymphomas in a subset of LANA-expressing transgenic mice [26]. These findings suggest that HHV8 lymphomagenesis requires additional genetic events and/or other viral/cellular oncoproteins.

In keeping with these observations, in MCD, the majority of the few microlymphoma cases described in the literature [4,5,15,27,28,29,30,31] were polyclonal (Table 2). Du et al. found two monoclonal unmutated (SHM 0.1–0.4%) microlymphomas out of eight cases [5]. Cloning and sequencing of the IGH PCR products confirmed the presence of a dominant clonal cell population in the two monoclonal microlymphomas, whereas multiple unrelated clones or multiple clones with one weakly dominant clone were detected in the six polyclonal microlymphomas. In addition, different microlymphomas from the same patient showed different clones, suggesting that these microlymphomas carried multiclonal B-cell populations with an uncertain malignant capacity. Indeed, two patients had concomitant nodal plasmablastic lymphoma, but only in one of them was the plasmablastic lymphoma clone present at a low frequency in the concurrent splenic microlymphomas. Of note, the other plasmablastic lymphoma case showed a monoclonal IGH but a polyclonal IGL genes rearrangement [5].

Similarly, we found a dominant IGH peak within a polyclonal background which corresponded to a 4% IGH clone by NGS analysis, but the presence of SHM in the rearranged IGHV gene was not compatible with a naïve B-cell origin. Likewise, the IGL gene was polyclonal, whereas the IGK gene showed a mild prominent peak, raising suspicion of early lymph node involvement by an EBV-neg ECPEL with a post-germinal center origin. In addition, gene scan analysis revealed in the bone marrow blood the same clone found in the lymph node, indicating that the clone was disseminated.

The fifth edition of the World Health Organization (WHO) Classification of Hematolymphoid Tumors (WHO-5) acknowledges that the distinction between nodal involvement by an EBV-neg ECPEL and HHV8+ DLBCL may be difficult [1], whereas the international consensus classification of mature lymphoid neoplasms (ICC) states that HHV8+ DLBCL should be favored in EBV-negative cases with cytoplasmic IgM, lambda, and/or those associated with MCD [32]. An aqssessment of clonality and SHM could help in this diagnostic dilemma, although, as in our case, it might make the distinction even more problematic, highlighting the possibility of a gray area between these two entities. The case described by Seliem et al. is also unusual [29]. An HIV+ patient showed aggregates of EBV+/HHV8+ plasmablasts in the germinal centers and within the sinuses of the lymph nodes. Although the polyclonality of the plasmablastic proliferation argued against an ECPEL favoring the diagnosis of a GLPD, the patient was HIV+, the lymph node showed features reminiscent of MCD, and the disease behaved aggressively. Two additional problematic cases of ECPEL versus HHV8+ DLBCL and GLPD have been reported recently by the 2022 European Association for Hematopathology/Society for Hematopathology lymphoma workshop, stressing the lack of consensus among expert hematopathologists in classifying such cases [33].

Mutational data are currently available for cases of PEL/ECPEL but not of HHV8+ DLBCL or monoclonal microlymphoma [9,33,34,35,36]. In our case of monoclonal HHV8+ microlymphoma associated with MCD, mutational analysis found a pathogenic mutation in *KMT2D* (alias *MLL4* or *MLL2*), a histone methyltransferase that regulates gene transcription and DNA repair [37,38]. *KMT2D* has been found to be recurrently mutated in diffuse large B-cell lymphoma (DLBCL) and follicular lymphoma (FL) [39] but not in PEL/ECPEL and in KS [9,13,33,34,35,36]. Additionally, we found VUS in the *KMT2A*, *FOXO1*, and *ARID1A* genes. Nonsynonymous mutations in these genes have also been described in DLBCL [40,41]. In particular, variants of the epigenetic genes *KMT2A* and *ARID1A* have been reported to enhance the correlation of *KMT2D* nonsynonymous mutations, with tumor genomic complexity and poor prognosis in wild-type *TP53* DLBCL [41], and to recur more frequently in EBV-positive DLBCL [42]. *FOXO1* encodes instead for a transcription factor coordinating the dark zone program required for the germinal center (GC) response. *FOXO1* variants, found in 15% of transformed FL and in 10% of de novo and 36% of relapsed or refractory DLBCL, are thought to favor the competitive expansion of *FOXO1*-mutant GC B-cells in the absence of synergistic signals, by activating programs resembling positive selection [43,44,45]. The mutations in *FOXO1*, *KMT2A*, and *ARID1A* we detected in our case did not perfectly match with those previously reported in the literature; thus, they did not support their definite pathogenicity. However, further studies focused on HHV8-driven lymphoproliferative disorders might better clarify their possible implication in this specific type of disease.

KS is also an enigmatic disease currently classified as a neoplasm, although its neoplastic nature is still debated, since contrasting data exist on clonality and genetic alterations [13]. The coexistence of MCD and KS in the same tissue is a common phenomenon. Naresh and colleagues [14] demonstrated that 63% of HIV-positive patients diagnosed as HHV8-MCD showed evidence of coexisting KS. They hypothesized that this association is due to the lytic HHV8 infection of B-lymphoid cells that expose endothelial cells to high levels of HHV8, resulting in the formation of KS tumorlets in the lymph nodes. In our report, the patient was initially diagnosed and treated for a cutaneous KS, and two months later developed a systemic lymphadenopathy, consistent with simultaneous involvement by HHV8-MCD and capsular micro-KS suggestive of a persistent HHV8 lytic infection.

## 4. Conclusions

In conclusion, we reported here the case of an HIV-negative patient who developed a rapidly fatal HHV8-driven multisystemic inflammatory syndrome, with concomitant lymph node involvement by KS, HHV8-MCD, and foci of IgMλ microlymphoma, in which IGH and mutational NGS analyses identified a monoclonal B-cell proliferation with a putative post-germinal center derivation and a pathogenic mutation in *KMT2D*. In the setting of a differential diagnosis between an initial HHV8+ DLBCL and an ECPEL, the integration of histopathological and molecular data highlighted, in our case, the difficulty of defining the possible evolution of the microlymphoma within the spectrum of HHV8-positive lymphoproliferations.

## Figures and Tables

**Table 1:** Somatic mutations detected by targeted NGS, including prediction of amino acid changes that affect protein function (SIFT, Polyphen2, mutation assessor, mutation taster).

Gene	Reference	Chr	cDNA	Coding Consequence	AAchange	COSMIC/ Clinvar	VAF	SIFT	Polyphen2	Mutation Assessor	Mutation Taster	Prediction
*KMT2D*	NM_003482	12q13.12	c.1940del	Frameshift	p.(Pro647fs)	COSV56415893/rs770315135	1.9	NA	NA	NA	NA	Pathogenic/Likely pathogenic
*KMT2D*	NM_003482	12q13.12	c.11843T>A	Missense	p.(Leu3948His)	COSV105187453/rs1943003450	5.5	Uncertain	Benign	Benign moderate (0)	Uncertain (1)	VUS
*KMT2D*	NM_003482	12q13.12	c.11756_11758del	Inframe_3	p.(Gln3919del)	COSV56474613/rs576788910	5.3	NA	NA	NA	NA	VUS
*KMT2A*	NM_001197104	11q23.3	c.46A>C	Missense	p.(Thr16Pro)	None	1.5	Uncertain (0.00)	Possibly damaging (0.914)	Benign moderate (0)	Benign supporting (0.99)	VUS
*FOXO1*	NM_002015	13q14.11	c.295G>C	Missense	p.(Ala99Pro)	None	3.3	Benign moderate (0.34)	Possibly damaging (0.652)	Benign moderate (0)	Uncertain (0.99)	VUS
*ARID1A*	NM_006015	1p36.11	c.64T>G	Missense	p.(Ser22Ala)	None	3.1	Pathogenic supporting (0.0)	Possibly damaging (0.956)	Benign moderate (0.55)	Benign moderate (0.69)	VUS

COSMIC: Catalogue of Somatic Mutations in Cancer; Chr: chromosome; AAchange: aminoacid change; VAF: variant allele frequency.

**Table 2:** Clinicopathological characteristics and clonality results of HHV8+ microlymphomas described in the literature.

Age/Sex	HIV Status	Diagnosis (Site of Involvement)	Plasmablasts Light-Chain Restriction	IGH Rearrangement	Associated Diseases (Site of Involvement)	Clinical Outcome
**Dupin et al. [4]**						
32/M	Positive	HHV8 MCD (LN, spleen) Microlymphoma (spleen)	Lambda	Unable to confirm monoclonality	KS (skin)	Death within 6 months from diagnosis of “plasmablast” crisis
62/M	Positive	HHV8 MCD (LN, spleen) Microlymphoma (spleen)	Lambda	Unable to confirm monoclonality	None	Death within 7 months from diagnosis of disease progression and ketoacidosis
47/F	Positive	HHV8 MCD (LN, spleen) Lymphoma (LN, spleen, pharynx) Microlymphoma (spleen)	Lambda	Unable to confirm monoclonality	KS (skin and palate)	Death within 9 months from diagnosis of plasmablastic lymphoma
**Du et al. [5]**						
-	Negative	HHV8 MCD (LN) Lymphoma (LN) Microlymphoma (spleen)	Lambda	Monoclonal	-	-
-	Positive	HHV8 MCD (LN) Microlymphoma (spleen)	Lambda	Polyclonal	-	-
-	Negative	HHV8 MCD (LN) Microlymphoma (LN)	Lambda	Polyclonal	-	-
-	Positive	HHV8 MCD (LN) Microlymphoma (spleen)	Lambda	Monoclonal	-	-
-	Positive	HHV8 MCD (LN) Lymphoma (LN) Microlymphoma (spleen)	Lambda	Polyclonal	-	-
-	Positive	HHV8 MCD (LN) Microlymphoma (spleen)	Lambda	Polyclonal	-	-
-	Positive	HHV8 MCD (LN) Microlymphoma (spleen)	Lambda	Polyclonal	-	-
-	Positive	HHV8 MCD (LN) Microlymphoma (spleen)	Lambda	-	-	-
-	Positive	HHV8 MCD Microlymphoma (LN)	Lambda	Polyclonal	-	-
**Li et al. [27]**						
61/M	Negative	HHV8 MCD (LN) Microlymphoma (LN)	Lambda	Polyclonal	Hemophagocytic Syndrome (BM)	Death within 1 month from diagnosis of acute respiratory distress syndrome
**Dargent et al. [28]**						
32/M	Positive	HHV8 MCD (LN) Microlymphoma (LN)	Lambda	Polyclonal	KS	Death shortly after diagnosis of multiple organ failure
**Eaton et al. [15]**						
42/M	Positive	HHV8 MCD (LN) Microlymphoma (LN)	Lambda	Not determined	KS (skin and LN)	Alive at 10 years follow-up
**Seliem et al. [29]**						
45/M	Positive	HHV8 MCD (LN) GLPD vs. Microlymphoma vs. ECPEL (LN and spleen)	Lambda (dim)	Polyclonal	KS (spleen and LN) Hemophagocytic syndrome (BM)	Death within 7 months from diagnosis of hemophagocytic syndrome
**Koenig et al. [30]**						
67/F	Negative	MCD (LN, spleen) Microlymphoma (LN)	Lambda	Not determined	-	Partial response to rituximab and complete response to siltuximab, alive
**Gonzalez-Farre et al. [31]**						
22/F	Positive	Plasmablastic-rich MCD (LN)	Lambda	Polyclonal	-	R-CHOP and intrathecal RT, alive 64 months after diagnosis
37/M	Positive	Plasmablastic-rich MCD (LN)	Lambda	Not determined	-	Death shortly after diagnosis
30/M	Positive	Plasmablastic-rich MCD (LN)	Lambda	Polyclonal	KS	R-CHOP and cART, alive 64 months after diagnosis
**Rogges et al.** (present case)						
66/M	Negative	MCD (LN) Microlymphoma (LN)	Lambda	Monoclonal	KS (skin and LN)	Death shortly after diagnosis of multiple organ failure

KS: Kaposi sarcoma; R-CHOP: Rituximab, cyclophosphamide, doxorubicin, vincristine, prednisone; cART: combined antiretroviral therapy.

## Data Availability

No new data were created or analyzed in this study. Data sharing is not applicable to this article.
